# Public psychology and holistic approaches to prevention and treatment of depression

**DOI:** 10.3389/fpsyt.2025.1600094

**Published:** 2025-06-03

**Authors:** Elissa H. Patterson, Chazlyn Miller, Madison Hannapel

**Affiliations:** ^1^ Institute for Healthcare Policy & Innovation, Consultation Liaison Psychiatry, Departments of Psychiatry and Neurology, University of Michigan Medical School, Ann Arbor, MI, United States; ^2^ Effects of Discrimination and Privilege Laboratory, Department of Psychology, Eastern Michigan University, Ypsilanti, MI, United States; ^3^ Consultation Liaison Psychiatry, Department of Psychiatry, University of Michigan Medical School, Ann Arbor, MI, United States; ^4^ Youth and Adolescent Relationships Laboratory, Department of Psychology, Eastern Michigan University, Ypsilanti, MI, United States

**Keywords:** depression, public psychology, prevention, treatment, quality of life, holistic, healthy behaviors, lifestyle factors

## Abstract

It has been known since ancient times that the mind, body, and social connection are intertwined to promote thriving. More specifically, the basic pillars of health and wellbeing have been described across cultures, and they are codified in modern healthcare as the principles of lifestyle medicine. However, across the globe, emotional despair, loneliness, and chronic diseases have been rising despite a wealth of knowledge about the elements that promote happy, healthy individuals and communities. Depression has been identified as a significant element of the global mental health crisis. We know that depression is a multifaceted condition influenced by biological, psychological, social, environmental, and spiritual factors, but prevailing medical models prioritize medication and overshadow the importance of the other facets. In healthcare, pharmaceutical companies comprise a major portion of innovation that has led to the development of invaluable life-saving medications. However, some psychiatric drug makers use marketing methods rather than the scientific method to test and promote the sale of drugs to treat depression. For example, a confidential data ownership and transfer agreement that came to light during litigation over an antidepressant drug, stated that the purpose of the data collected by the drug company sponsored research was to market their product. The public and the medical community have been led to believe that drugs are more scientifically valid than other approaches to depression. We suggest a more holistic approach to prevention and treatment of emotional despair and depression, an approach that uses a public psychology lens to promote societal wellbeing.

## Introduction

Societal levels of emotional despair and chronic illnesses are on the rise (1). High levels of burnout, job dissatisfaction (2), discrimination, emotionally polarized political discourse (3), hate crimes (4), social/digital media violence (5, 6), civil unrest (7), unhealthy physical environments (1), loneliness (8), food insecurity, and unhealthy foods all contribute to decreases in wellbeing (9).

Yet, sadness or loss of pleasure that lasts for two weeks, regardless of socio-environmental context, is one of the main criteria for diagnosing a biomedical brain disease called depression [Major Depressive Disorder, according to psychiatric classification systems (10, 11)]. Considering the social context of increasing despair, public psychology (12)—the application of psychological knowledge to address real-world social problems—invites an ecological interpretation of depression as a condition that emerges from complex interactions between individuals and their environments, rather than an individual brain disease. This perspective aligns with evidence that depression’s etiology spans biological, psychological, social, environmental, and spiritual domains.

The biomedical disease model of depression has been bolstered by advertising campaigns promoting the “chemical imbalance” theory (13) and misleading summaries of research findings. This includes clinical trials designed for marketing and selling drugs, not for identifying whether or not a drug is helpful to patients. For example, litigation about the antidepressant drug Zoloft surfaced a confidential data ownership and transfer agreement that explicitly stated that the purpose of the data collected through the drug company sponsored research was to “support directly or indirectly, marketing of our product” (14). These drug promotion campaigns have effectively overshadowed data showing that other interventions and placebos perform on par with, if not better than drugs, especially considering potentially life-threatening drug side effects (15–19). A placebo is an inert substance that is given to a patient as a comparator to test the effects of a new treatment. According to what used to be canonical theory, a placebo should not lead to improvement in a patient’s condition compared to an active treatment, but it is clear now that placebos themselves do lead to improvements. Studies of the natural history of depression and corrective reanalysis of pharmaceutical company data raise questions about the validity of the presumed superiority of the biomedical drug-treatment model of depression (20–24).

Furthermore, the standard medical treatment for depression—antidepressant drugs—carries a black box warning about increased suicidal thinking and behaviors in the US and is restricted for children in Europe (20, 25, 26). Paradoxically, the suicidality risk associated with antidepressants may become a justification for more invasive interventions like electroconvulsive therapy (ECT), purportedly to prevent suicide (27). While rapid improvement in some ECT patients can create the impression of miraculous effectiveness, the reality is more complex. Escalating treatment to ECT presents significant risks, as some patients emerge from multiple ECT sessions still depressed but with added cognitive and memory impairments, along with small but increased mortality risks from repeated anesthesia exposure (28–30). Research increasingly suggests that many depression treatments—including both medications and ECT—may work through placebo effects, hope, healing rituals, expectations, and therapeutic relationships rather than their alleged mechanisms (16, 31, 32).

Emerging scientific research on principles of healing and holistic approaches to prevention and treatment of depression demonstrate numerous methods to promote mental health, with minimal side effects (33). Rather than narrowly attempting to repair isolated components, holistic approaches evaluate and enhance the performance of the entire system. These methods are grounded on the premise that human health and wellbeing require systematic balance among certain elemental building blocks and that symptoms of depression surface when one or more of those elements is disrupted. To maximize effectiveness, the remedies must be tailored to the type of disruption and patient preferences. Many options are free, readily accessible, and offer patients, long-lasting benefits. On the other hand, cost or lack of access to many of these approaches can compound the difficult challenge of identifying which interventions are most worthwhile for a specific individual or community. In addition, it takes greater effort to engage with these non-pharmacological aspects of health promotion than it does to take a pill, as one might if following a typical biomedical approach.

While acknowledging that pharmaceutical interventions have helped many individuals, this paper presents a broader approach to depression prevention and treatment grounded in an integrative, holistic framework. We outline the foundations of wellbeing, then strategies for developing environments that actively foster whole health. We emphasize how collective action and systemic change must complement individual-level interventions to create meaningful prevention and treatment approaches to depression and overall wellbeing. And finally, we explore a wide range of methods to foster wellbeing depending on an individual’s values, preferences, and access. Together, these approaches constitute a holistic framework aimed not only at reducing the risk of depression but also at promoting and supporting overall thriving and resilience.

## Biopsychosocial, environmental, and spiritual determinants of wellbeing

There has been much written elsewhere about the neuroscientific and genetic correlates of depression, and it is clear that there is a bidirectional influence between behavior and the brain. Here we shift focus toward practical variables that are central to solving the mental health crisis at both individual and communal levels which are often underemphasized in conventional medical treatment approaches.

The emerging field of lifestyle medicine provides a framework based on pillars that strengthen the drivers of whole person health. These pillars include adequate physical movement, sleep, plant-based whole food nutrition, social connection, stress-management, and avoiding harmful substances (34). Beyond these fundamental elements, a sense of meaning and purpose provides essential motivation that empowers individuals to persevere through life’s challenges (35). And spirituality, defined here as connection to something greater than oneself, represents another dimension linked to psychological resilience and thriving (36).

The origins of lifestyle medicine pillars are evident in Traditional Chinese Medicine (TCM) and Ayurvedic medicine, both of which focus on strengthening the whole person by balancing energy rather than reacting to reductionist aspects of illness. TCM can involve nutritional advice, psychological management and education, acupressure, exercise, and regulation of the sleep-wake cycle. A meta-analysis including 18 studies demonstrated promising results from TCM interventions used to relieve depression symptoms, though the authors note that the literature could be improved by additional studies with higher quality methods (37). Similarly, Ayurvedic Medicine protocols have been created to address the biomedical concept of depression through a combination of mind-body exercise, counselling, ayurvedic drugs, mantras and music, with good results (38).

However, there is not a clear universal path to strengthen lifestyle promoters of wellbeing, given the myriad cultural, cognitive, emotional, social, and personality factors that sway an individual’s behavior both above and below conscious awareness.

## Healing mechanisms

Psychological health determines how effectively one’s inner world supports or impedes their ability to thrive. Drawing from positive psychology, it is known that psychological wellbeing encompasses not just the absence of distress, but the presence of strengths, virtues, and positive emotions that foster resilience and meaning (39). The core components include optimistic thought patterns, emotional intelligence, nurturing social connections, and a sense of purpose through belonging. Consider: Can an individual think with clarity, leverage their character strengths, cultivate positive emotions, and form meaningful relationships that contribute to their growth? Psychotherapists and other clinicians help their clients achieve a state of mind that makes it easier to engage in health-promoting behaviors through various therapeutic schools of thought. There are hundreds of types of psychotherapy, but research shows that therapeutic outcomes depend less on the specific type of therapy than on “common factors”—including therapeutic alliance, empathy, positive regard, a culturally relevant narrative, positive expectations, and shared goals (40).

These common factors align with core healing mechanisms identified in placebo research and ancient medicine before that. Contemporary science has reframed our understanding of placebos–they are not “fake” treatments that produce imaginary results, but powerful catalysts that activate innate healing capacities through hope, ritual, resilience, and sophisticated pathways (41–44). They trigger conditioned physiological responses, regulate the autonomic nervous system, modulate the hypothalamic-pituitary-adrenal axis, and provide protected space for natural recovery processes to unfold without interference (45–47).

This understanding has profound implications for depression treatment. When patients internalize the biomedical narrative that depression is fundamentally a brain disease requiring chemical intervention, their hope for treatment can diminish (48). When antidepressants—which demonstrate meaningful benefits in only a subset of patients—fail to work, individuals who have accepted the brain disease model are prone to conclude [erroneously] that their condition is intractable or that they themselves are somehow irreparably broken (49). This iatrogenic hopelessness is particularly troubling when we consider the evidence for natural recovery: one striking study demonstrated that 85% of individuals experiencing depression recover within one year without formal treatment (50). This suggests that some current treatment approaches, by promoting limiting beliefs about the nature of depression, may actually interfere with the natural processes that facilitate recovery for many people.

The prevalence of individuals with what the biomedical model refers to as treatment resistant depression has led to a resurgence of medical interest in using psychedelic plants and chemicals to alter consciousness and expand cognitive flexibility as a treatment for depression. This approach may be particularly valuable for individuals who have been iatrogenically entrenched in neurochemistry-altering drug treatment and rigid thinking about the nature of depression as a brain disease (24). Shamanic and indigenous healing traditions included the use of psychedelics long before the Western medicine community noticed their potential (51, 52). Advocacy efforts to decriminalize these psychedelics are an example of how cultural humility combined with legal and policy changes can open access to new avenues for prevention, treatment, and healing.

## Building environments that promote public wellbeing

Environmental factors such as the stressors of poverty, access to education and childcare, institutionalized discrimination and oppression, food deserts, pollution, social isolation, and violence present significant challenges to optimizing the multiple domains that determine wellbeing. Therefore, designing environments to facilitate health-promoting behaviors can improve community health (53, 54). For example, an individual might engage in more healthy physical activity if their city has a safe, well-lit, inviting community-oriented green space for walking or bicycling (55). Similarly, an individual may eat healthier foods if they live in a neighborhood with zoning regulations and commercial incentives that nurture the success of well-stocked grocery stores within walking distance, rather than fast food restaurants with low-priced high-fat, high-sugar foods that promote obesity and type 2 diabetes (56). Additionally, municipalities that sponsor complimentary programming, such as senior centers and community gardens or activities in public parks may cultivate greater social cohesion. Affordable housing, well-resourced schools, accessible childcare, and jobs providing a living wage significantly reduce daily stressors. Economic and political conditions that support robust civic infrastructure make it easier for individuals to live, work, experience joy, rest, sleep, and engage meaningfully with others, creating conditions that foster overall flourishing and wellbeing (57).

The influences of health-promoting external factors are so powerful that researchers have identified them in “Blue Zones”—communities where people, on average, are believed to live longer, happier, healthier lives (58). The distinguishing behaviors in these communities mirror the principles of lifestyle medicine: incorporating natural movement into daily living, eating primarily plant-based whole foods, taking time for relaxation, connecting meaningfully with others, and living with a sense of purpose. These principles can guide municipalities that aspire to engineer better health outcomes by building supportive infrastructure. Public health experts, policy makers, urban planners, and social justice activists can all advocate for implementation of these principles.

Workplace culture can inadvertently encourage unhealthy competitive, self-sacrificing, or workaholic behaviors that profoundly influence mental health. There is now even a scale to measure ‘rest intolerance’—the negative self-worth and emotions experienced when resting (59). On the other hand, organizations can generate successful, socially satisfying environments starting with leadership that models reliability, transparent communications about expectations and accountability, psychological safety, opportunities for growth, and responsiveness to the needs of employees (60).

Decision-making autonomy, social support, and job security are also implicated in workplace factors that promote or inhibit psychological wellbeing (61). Despite evidence-based approaches to improve work environments (62), problematic organizational cultures remain widespread, with more than half of workers reporting significant work-related stress in one survey (2). Prolonged stress manifests as burnout, a topic of growing importance in healthcare, given high levels among physicians (63), medical students (64) and other staff, particularly with the compounded rise of moral injury during and after the COVID-19 global pandemic (65, 66). When psychological distress emerges in these contexts, it represents a predictable response to adverse conditions rather than an individual failing. Yet the framework of the medical model inherently places responsibility on the employee rather than recognizing the role of toxic environments (67).

## Integrative wellbeing

### Mind-body regulation

The scientific era of modern medicine has produced awe-inspiring breakthroughs in treating human ailments. However, this progress has been accompanied by an increasingly reductionist approach to evidence that unintentionally devalues the less tangible dimensions of healing. Mind-body interventions—practices that have been integral to healing traditions since antiquity—often fall victim to this narrowed perspective despite their enduring therapeutic value. These practices promote balanced integration between the mind and the body, and include breathing techniques, intentional movement, meditation, chanting, the use of sound, and rhythm. Their benefits are described within the social networks that use them, and there is significant scientific evidence mounting as well. Mind-body integrative activities such as qigong, tai chi, yoga, mindfulness meditation, aromatherapy, music engagement (68), autonomous sensory meridian response (ASMR) (69) and sexual activity (70, 71) demonstrate utility in improving mental health (72, 73). While no single activity is likely to benefit everyone, these activities are examples of methods that individuals can choose to elicit restorative moments. It is worth noting that randomized controlled trials, which are typically considered the gold standard for testing healthcare treatments, cannot always detect benefits of interventions to which individuals react idiosyncratically. For example, some people might feel relaxed and soothed by yoga, and others might experience distressing self-judgment. Statistically, when there is a wide range of responses to a given intervention, including positive and negative responses, there is a heterogeneity of treatment effects that may lead to a type 2 error (i.e. the relaxation benefits of yoga could be undetectable because the positive and negative effects cancel each other statistically). This can lead to the erroneous dismissal of valuable interventions (74).

Beyond benefits on mood and relaxation, mind-body practices like those above enhance sleep quality (73) —a critical factor in mental health maintenance (75). These practices can be part of sleep hygiene rituals to ease the transition from wakefulness to deep sleep, which global prevalence estimates suggest is a struggle for 16.6% - 70% of people, depending on the population studied (76, 77). Poor sleep affects all aspects of health including the cardiovascular system, reproductive system, cognition, memory, and insulin-regulating systems implicated in the development of type 2 diabetes (78, 79).

Further solidifying its vital role, sleep disruption triggers proinflammatory cytokines associated with core depressive symptoms: anhedonia, social withdrawal, hyperalgesia, appetite dysregulation, and fatigue (75, 80). This inflammatory response pattern, termed Sickness Behavior, was originally described in the context of a cold or flu, but has since been found to be relevant in other circumstances (81). It represents an evolutionarily conserved protective mechanism that redirects energy resources toward immune function when pathogens are detected. Proinflammatory cytokines are also elevated acutely after surgeries and traumatic injuries, and can remain elevated for weeks, compounding the emotional challenges of patients during recovery (82).

Sleep-wake architecture is governed by intricate circadian fluctuations in neurotransmitters and hormonal networks that are influenced by the environment and the amount of time a person has been awake. These systems can become dysregulated during acute and prolonged periods of stress and depressive episodes (75). Among many available interventions, light therapy can help normalize circadian rhythms to promote resilience. Light therapy provides external zeitgebers that recalibrate disrupted neurotransmitter and hormonal systems, effectively reducing depressive symptoms including persistent sadness and fatigue (83). While light therapy and interventions to improve sleep might be extremely beneficial for one person, these interventions are not the solution for everyone, due to individual differences.

### Physical activity

The World Health Organization has issued a strong recommendation for adults to incorporate physical activity into weekly routines to improve mental health, cognition, cardiovascular health, and sleep, decrease mortality, decrease the incidence of type-2 diabetes and certain types of cancer, and better regulate adiposity (84). Exercise effects mood directly and also indirectly by diminishing the burden of chronic illnesses through numerous physiological and psychological mechanisms. Exercise benefits mental health by regulating glucose levels, the immune system, promoting insulin sensitivity, stimulating endorphin release, promoting brain derived neurotrophic factor, regulating cortisol levels and moderating stress and anxiety responses (85–87). Psychologically, it boosts mood, promotes self-esteem, emotional resilience, and deep states of relaxation (88).

A large-scale systematic review and network meta-analysis demonstrated that exercise is as effective at relieving depression as medication or counseling (89). The observed effect sizes were strongest for walking or jogging, strength training, and yoga. As with mind-body practices that promote wellbeing, there are many different types of physical movement to suit different circumstances, preferences, and values. Qigong and tai chi are two of the oldest known systematic physical exercises for wellness and balancing energy flow through the body, and numerous recent studies demonstrate their benefits for decreasing symptoms of depression (90).

Research suggests that both excessive and insufficient exercise diminish these potential benefits (91). While exercise serves as a powerful first-line intervention, certain circumstances exist in which it leads to malaise rather than mood improvement (92). Though a universally applicable exercise prescription remains unlikely, the optimization of movement should be integrated as a routine component of depression prevention and treatment protocols.

### Nature and green spaces

Throughout history, the healing power of nature has been recognized across diverse human traditions. This belief appears in TCM, Ayurvedic practices, Ancient Egyptian medicine, shamanic rituals, and indigenous healing systems worldwide (51). Human interconnectedness with nature and with natural forces that promote healing or illness were also part of Ancient Greek medical principles. In Japan, the term shinrin-yoku refers to the health-promoting practice of immersing oneself in the stimuli of the natural world (93). And in Scandinavian countries, the term friluftsliv, translated as “free-air life” refers to the life-enriching opportunities for growth offered by experiences in nature (94).

The quest to scientifically understand the restorative effects of nature has led to physiological and psychological evidence confirming nature’s influence. Studies demonstrate increased positive mood as well as increased parasympathetic and decreased sympathetic nervous system activity evoked when participants are in nature (94). Plant-derived volatile organic compounds promote relaxation, enhance immune function and anti-inflammatory activity in the body. Exposure to these compounds in both natural and controlled environments has been related to improved cognitive performance and mood regulation, underscoring their therapeutic potential (95, 96). These effects may stem from an innate physiological response or a conditioned association with the sights, sounds, and scents of nature that can evoke relaxation and comfort in some people (97, 98).

Beyond individual benefits, environmental interventions can significantly impact mental health at the population level. Research on urban green spaces indicates that greater access to nature is associated with lower depression rates among residents, reinforcing the importance of incorporating green spaces into city planning to promote mental well-being (99).

### Nutrition

Dietary approaches to promote wellbeing are prominent in both traditional and modern systems of healing (38). Nutritional deficiencies have been identified as an intervention target in depression treatment, though the research is not entirely consistent. The challenge of identifying universally applicable dietary guidelines using randomized controlled trials remains significant. If nutrition parameters are measured, the etiological heterogeneity combined with individual metabolic differences may still obscure patterns in complex data. Nonetheless, there are numerous micronutrients, foods, and herbal supplements under study. For example, it is known that the brain functions best with optimal amounts of omega-3 fatty acids, B vitamins, vitamin D, zinc, selenium, iron, calcium, and magnesium (100). Traditional herbal and plant supplements are also under study using modern scientific methods to identify the active ingredients that promote mental wellbeing (101).

The field of nutritional psychiatry has emerged recently to explore connections between diet, gut health, and mental wellbeing. Data demonstrates that stress and dietary patterns shape microbial composition and neuroinflammatory pathways linked to depression (102, 103). The gut-brain axis—bidirectional communication between the gut microbiome and central nervous system—plays a key role in mood regulation and cognition. Some forms of depression are linked to gut microbiota imbalances that trigger inflammation and stress responses (104). Diets that are high in processed foods contribute to gut dysbiosis and neuroinflammation, while anti-inflammatory diets like the Mediterranean diet have been shown to promote gut health and correlate with reduced depressive symptoms (102, 105). Highly refined foods and sugars contribute to greater fluctuations in glycemic index, which is also associated with disruptions in mood (106). Clinical evidence substantiates the use of personalized Mediterranean and plant-based diets to lower inflammation and improve mental health outcomes (103). Additionally, there is evidence that probiotic supplementation, particularly with *Lactobacillus* and *Bifidobacterium* strains can enhance microbial diversity and reduce depression-linked inflammation (102, 104). Dietary recommendations that are beneficial for emotional health are also beneficial for the body and associated with decreased morbidity and mortality (107). These findings highlight the need to integrate nutritional approaches into individual treatment and public health initiatives, promoting access to healthy foods and education on diet’s role in mental health.

## Conclusion: a holistic framework for depression prevention and treatment

Our examination of depression through multiple lenses reveals the limitations of reductionist approaches that frame depression through a biomedical lens. Depression arises from the complex interplay among biological, psychological, social, environmental, and spiritual factors. In contrast to framing depression solely as a brain disease—which places responsibility for change primarily at the individual level—this holistic approach expands intervention possibilities and encourages comprehensive solutions. Viewing depression through this integrative lens allows for the identification of diverse pathways that actively foster flourishing, resilience, and overall wellbeing.

The challenges in shifting from a medicalized to a holistic model of depression treatment stem from systemic, educational, and pharmacological factors. While evidence supports integrative approaches, decades of clinical practices and misconceptions create barriers to widespread adoption.

### Systemic and educational barriers

Entrenched medical model frameworks underpin most mental health and medical training programs, reinforcing the erroneous belief that antidepressant drugs repair neurotransmitter networks that are purportedly biologically broken. This creates a workforce less confident about the power of non-pharmacological approaches, both in implementation logistics and perceived effectiveness. Institutional infrastructure reinforces this paradigm through insurance reimbursement policies favoring pharmaceutical fixes, diagnostic frameworks prioritizing symptom suppression over root-cause analysis, and public expectations shaped by direct-to-consumer advertising of antidepressants.

### Pharmacological considerations

Antidepressants exert varied biological effects that can mimic holistic recovery pathways. For example, sleep promotion, increased energy, and increased appetite. Emotional blunting is also reported by patients. While these drugs may provide short-term functional improvements for some patients, their effects are nonspecific and achievable through other modalities without common side effects like sexual dysfunction or emotional numbing.

The transition to integrative care requires simultaneous reforms in medical education, insurance structures, and public health messaging to address depression’s multifactorial nature. Current systems remain focused on access to pharmaceutical interventions rather than sustained recovery through lifestyle, social, and environmental adjustments (36).

Addressing the global mental health crisis requires a paradigm shift from medicalized approaches toward holistic models that promote flourishing across multiple domains. This shift demands collaboration across disciplines—bringing together insights from public health, urban planning, mental health disciplines, lifestyle medicine and mind-body traditions of healing to create conditions where wellbeing can naturally emerge.

Moving forward, several principles should guide our collective response:

1. **Embrace complexity**: Effective interventions must acknowledge depression arises from interactions between biological vulnerabilities, psychological processes, social relationships, environmental conditions, and spiritual dimensions.
2. **Prioritize prevention**: By designing communities, workplaces, institutions and policies that naturally foster whole health, we can decrease depression incidence while enhancing quality of life.
3. **Integrate healing mechanisms**: Common factors in psychotherapy and biological underpinnings of placebo responses point toward universal healing mechanisms that can be activated through diverse modalities. We can implement these elements of healing into whichever treatment and prevention strategies are available in any given social context.
4. **Respect individual variation**: While foundational elements of wellbeing remain consistent, specific interventions that benefit individuals vary significantly based on cultural norms and other social determinants of health.
5. **Challenge limiting narratives**: Narratives that emphasize capacity for healing, resilience, and environmental determinants of health may better support recovery than the biomedical disease model.

If an urban planner suggests incorporating public art displays and green spaces into a new development, we can embrace this initiative as a valid form of prevention consistent with the complex etiology of depression. Similarly, publicly displayed art in green spaces serves as a preventive measure that incorporates nature, social bonding, physical movement, and connection to something larger than oneself.

Focusing on the functions served by various interventions is more important than which activity a person chooses to engage with. The heterogeneity of clinical responses combined with a rigorous framework for understanding factors that promote healing make it clear that mental health prevention and treatment can be greatly expanded and still be well within the bounds of contemporary scientific credibility. For instance, tai chi and weightlifting both involve mind-body integration and can be credible elements of a holistic plan, depending on social context. By maintaining openness to these variations and the value of non-biomedical interventions, we can ensure inclusivity and effectiveness in our collective approach to the promotion of thriving.

## Data Availability

The original contributions presented in the study are included in the article/**Supplementary Material**. Further inquiries can be directed to the corresponding author.
