# Supplementary material for: Public psychology and holistic approaches to prevention and treatment of depression
Source: Front Psychiatry. 2025 Jun 3;16:1600094. doi: 10.3389/fpsyt.2025.1600094 (PMC12171956; doi:10.3389/fpsyt.2025.1600094)
Supplement: Supplementary file 1 [file Presentation1.pptx]

## Slide 1
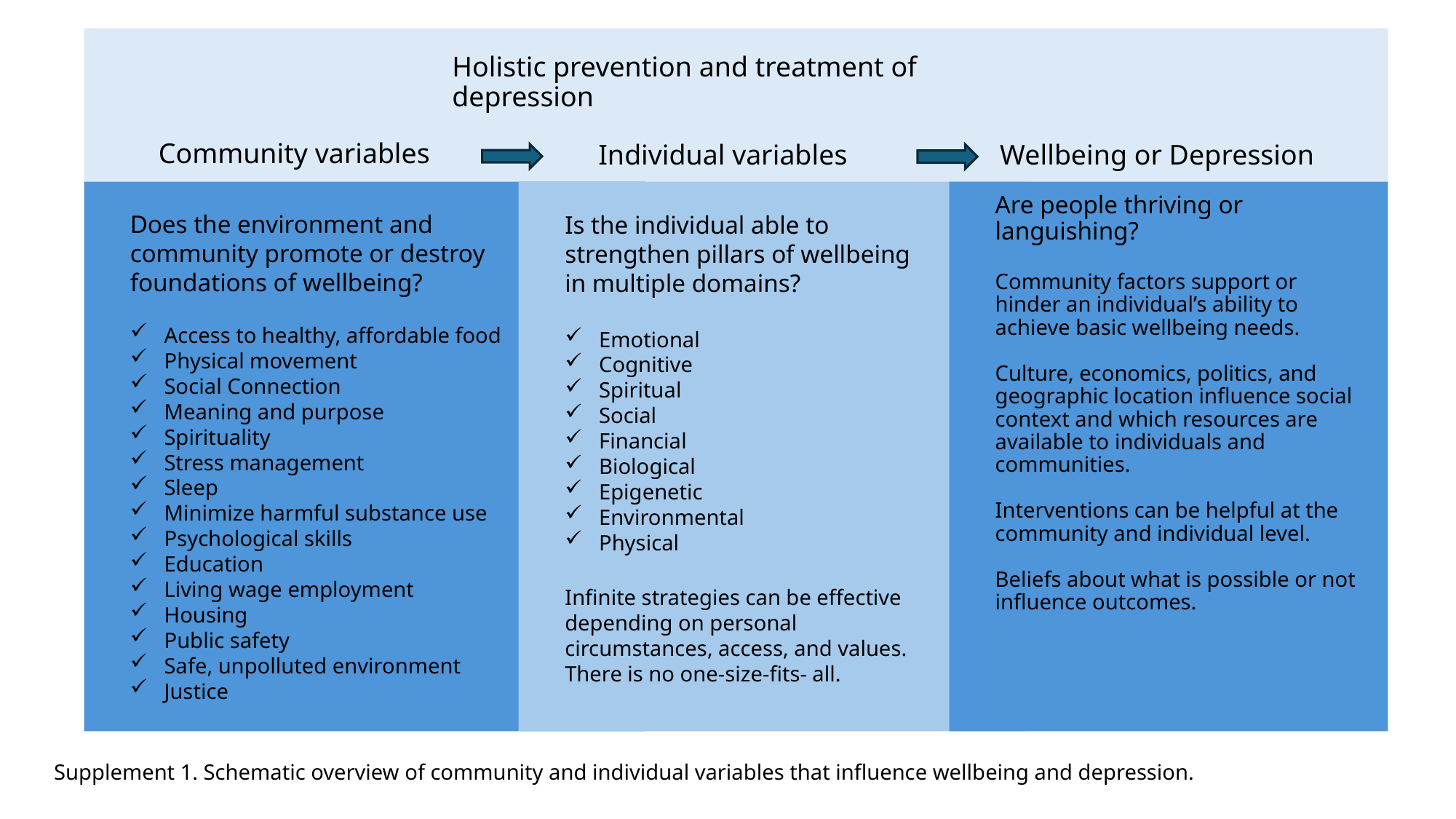

# Holistic prevention and treatment of depression
Community variables
Individual variables
Wellbeing or Depression
Does the environment and community promote or destroy foundations of wellbeing?
Access to healthy, affordable food
Physical movement
Social Connection
Meaning and purpose
Spirituality
Stress management
Sleep
Minimize harmful substance use
Psychological skills
Education
Living wage employment
Housing
Public safety
Safe, unpolluted environment
Justice
Are people thriving or languishing?
Community factors support or hinder an individual’s ability to achieve basic wellbeing needs.
Culture, economics, politics, and geographic location influence social context and which resources are available to individuals and communities.
Interventions can be helpful at the community and individual level.
Beliefs about what is possible or not influence outcomes.
Is the individual able to strengthen pillars of wellbeing in multiple domains?
Emotional
Cognitive
Spiritual
Social
Financial
Biological
Epigenetic
Environmental
Physical
Infinite strategies can be effective depending on personal circumstances, access, and values. There is no one-size-fits- all.
Supplement 1. Schematic overview of community and individual variables that influence wellbeing and depression.

## Slide 2
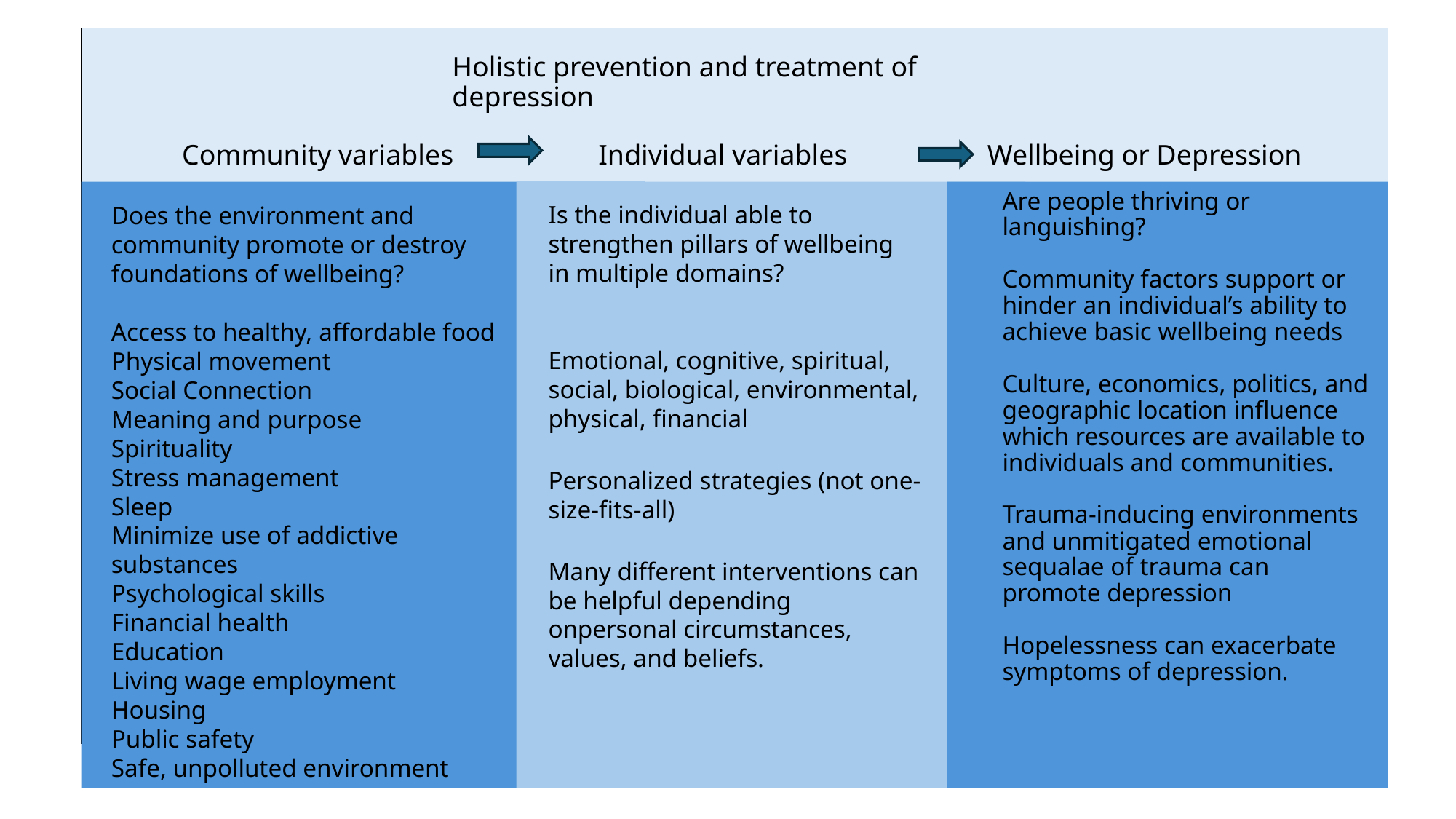

# Holistic prevention and treatment of depression
Community variables
Individual variables
Wellbeing or Depression
Are people thriving or languishing?
Community factors support or hinder an individual’s ability to achieve basic wellbeing needs
Culture, economics, politics, and geographic location influence which resources are available to individuals and communities.
Trauma-inducing environments and unmitigated emotional sequalae of trauma can promote depression
Hopelessness can exacerbate symptoms of depression.
Is the individual able to strengthen pillars of wellbeing in multiple domains?
Emotional, cognitive, spiritual, social, biological, environmental, physical, financial
Personalized strategies (not one-size-fits-all)
Many different interventions can be helpful depending onpersonal circumstances, values, and beliefs.
Does the environment and community promote or destroy foundations of wellbeing?
Access to healthy, affordable food
Physical movement
Social Connection
Meaning and purpose
Spirituality
Stress management
Sleep
Minimize use of addictive substances
Psychological skills
Financial health
Education
Living wage employment
Housing
Public safety
Safe, unpolluted environment

## Slide 3
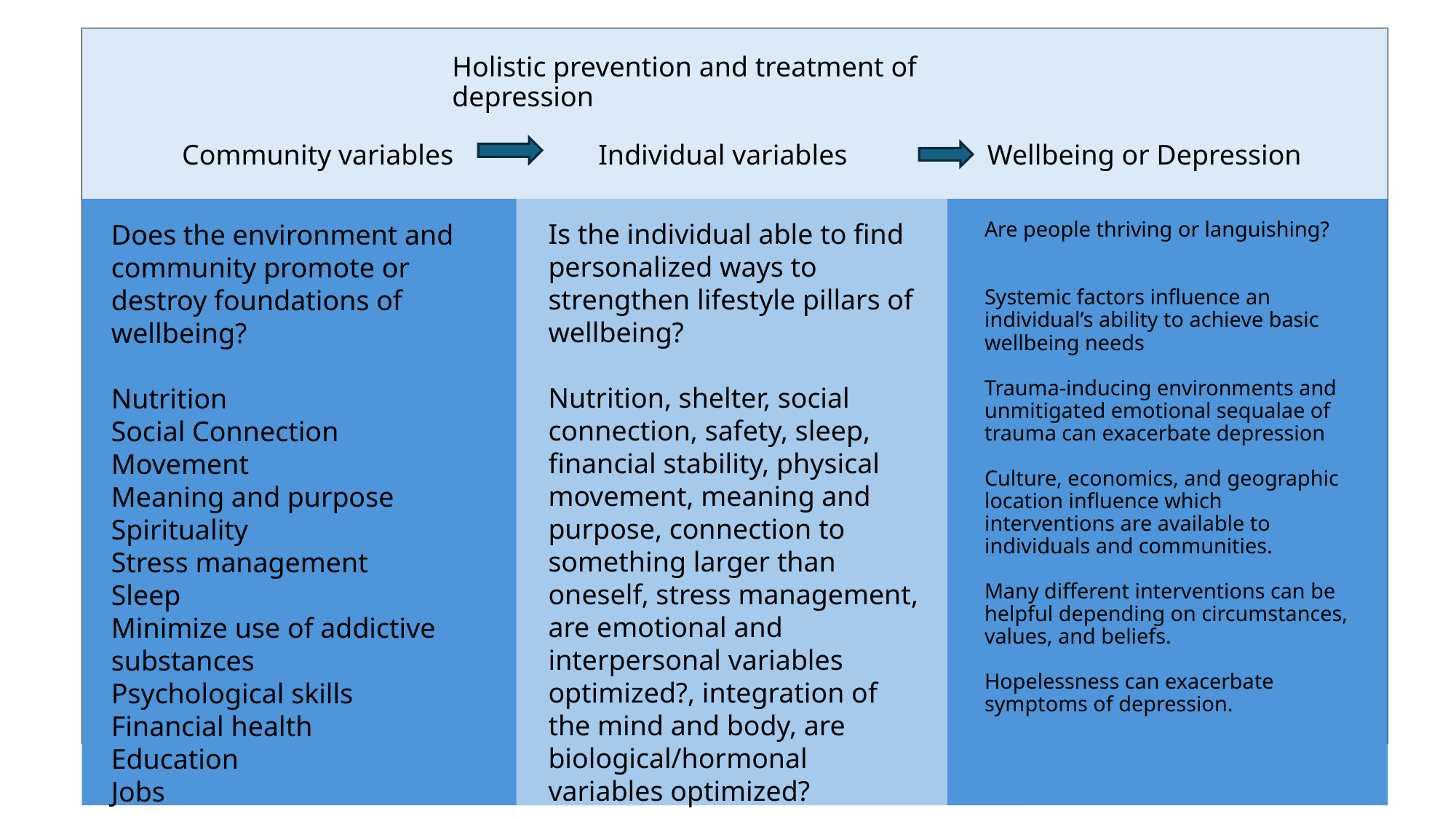

# Holistic prevention and treatment of depression
Community variables
Individual variables
Wellbeing or Depression
Are people thriving or languishing?
Systemic factors influence an individual’s ability to achieve basic wellbeing needs
Trauma-inducing environments and unmitigated emotional sequalae of trauma can exacerbate depression
Culture, economics, and geographic location influence which interventions are available to individuals and communities.
Many different interventions can be helpful depending on circumstances, values, and beliefs.
Hopelessness can exacerbate symptoms of depression.
Is the individual able to find personalized ways to strengthen lifestyle pillars of wellbeing?
Nutrition, shelter, social connection, safety, sleep, financial stability, physical movement, meaning and purpose, connection to something larger than oneself, stress management, are emotional and interpersonal variables optimized?, integration of the mind and body, are biological/hormonal variables optimized?
Does the environment and community promote or destroy foundations of wellbeing?
Nutrition
Social Connection
Movement
Meaning and purpose
Spirituality
Stress management
Sleep
Minimize use of addictive substances
Psychological skills
Financial health
Education
Jobs
Public safety vs crime/war, access to green spaces, walkable communities that foster social interactions, access to affordable health-promoting foods, living-wage jobs, civility, public services (education, infrastructure, non-polluted environments, access to healthcare, equitable justice system),

## Slide 4
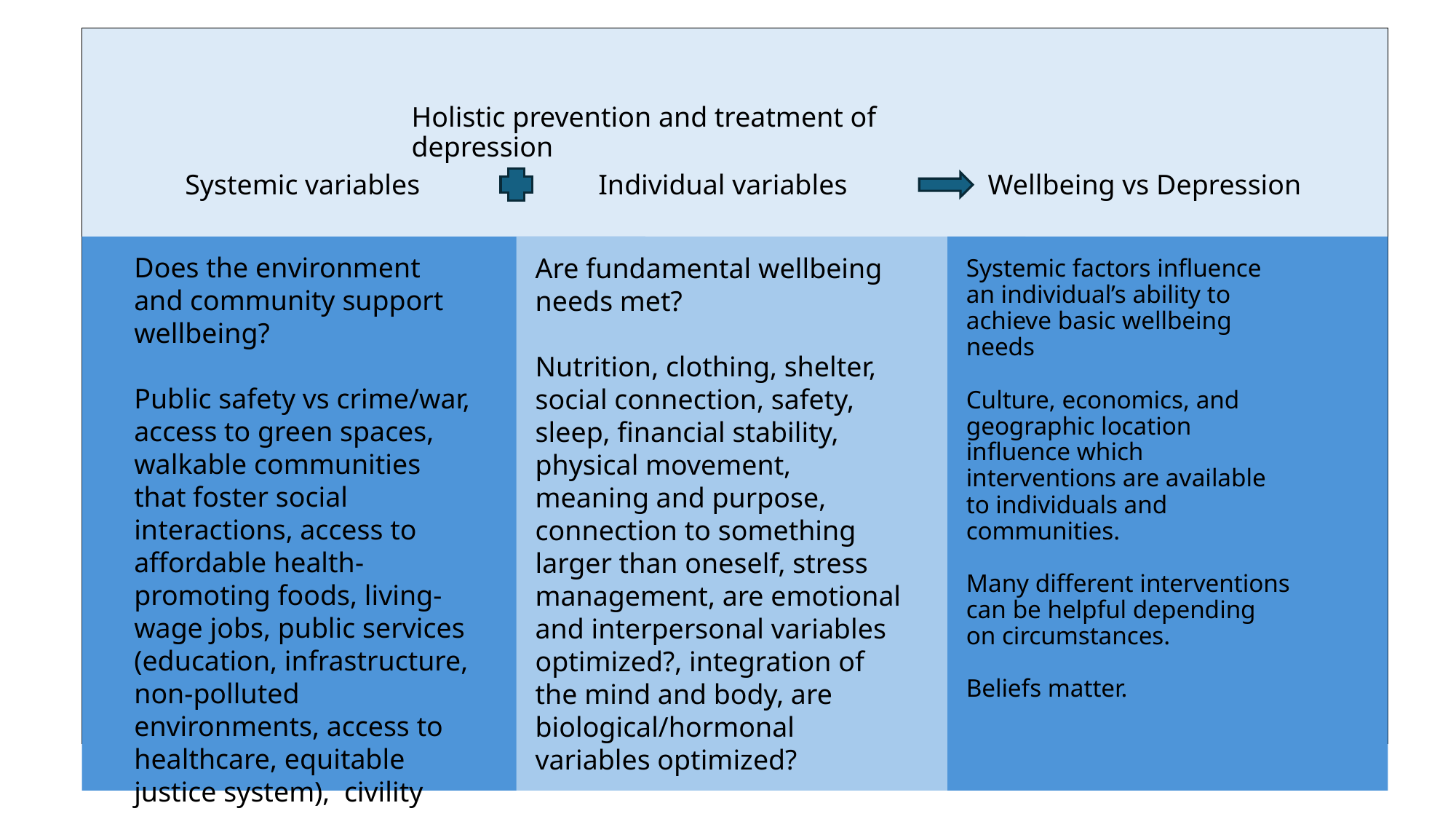

# Holistic prevention and treatment of depression
Systemic variables
Individual variables
Wellbeing vs Depression
Does the environment and community support wellbeing?
Public safety vs crime/war, access to green spaces, walkable communities that foster social interactions, access to affordable health-promoting foods, living-wage jobs, public services (education, infrastructure, non-polluted environments, access to healthcare, equitable justice system), civility
Are fundamental wellbeing needs met?
Nutrition, clothing, shelter, social connection, safety, sleep, financial stability, physical movement, meaning and purpose, connection to something larger than oneself, stress management, are emotional and interpersonal variables optimized?, integration of the mind and body, are biological/hormonal variables optimized?
Systemic factors influence an individual’s ability to achieve basic wellbeing needs
Culture, economics, and geographic location influence which interventions are available to individuals and communities.
Many different interventions can be helpful depending on circumstances.
Beliefs matter.

## Slide 5
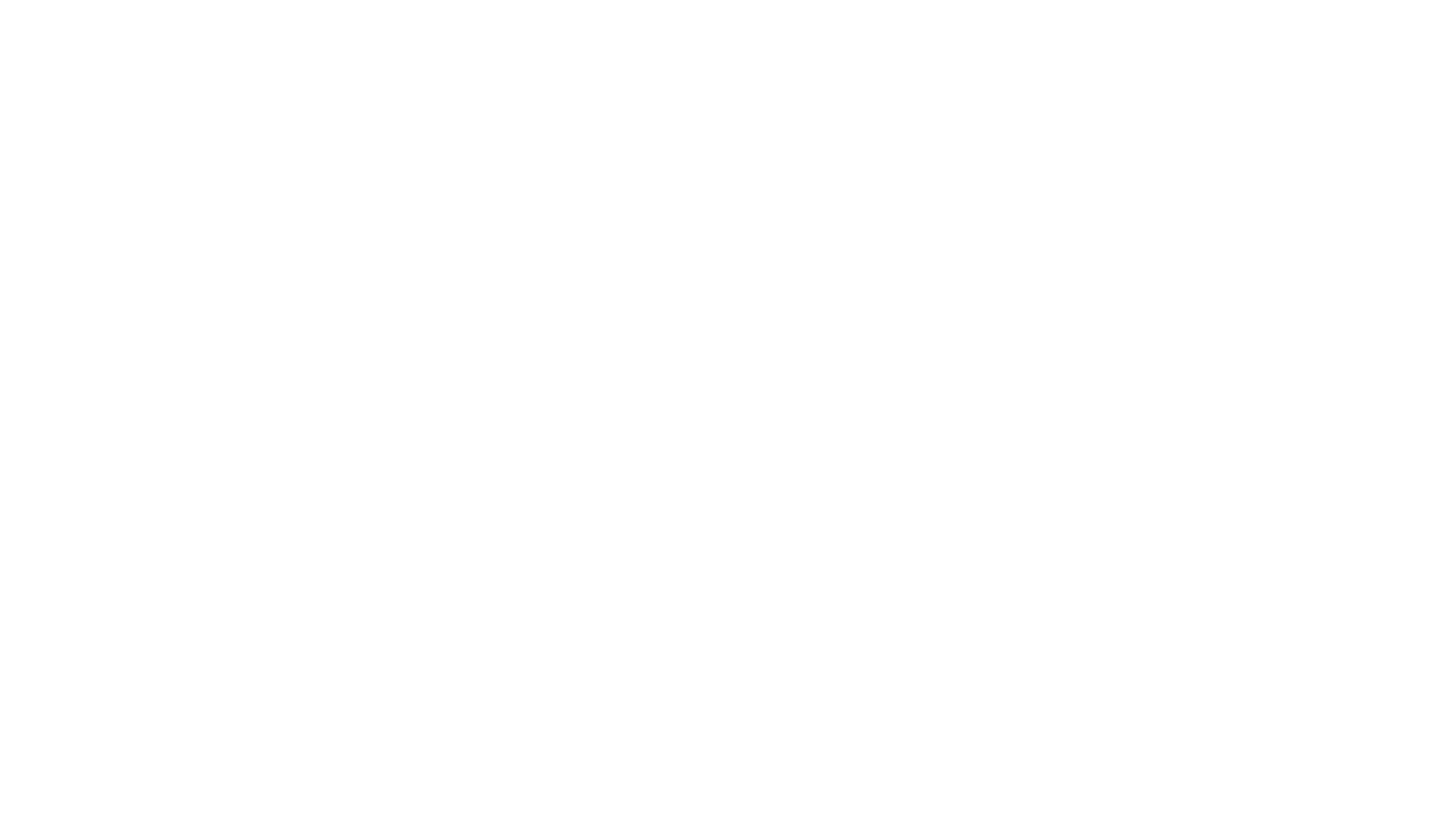

#
